# Hyposalivation in autoimmune diseases

**DOI:** 10.1007/s00296-012-2611-1

**Published:** 2012-12-29

**Authors:** Etsuko Maeshima, Kanako Furukawa, Shinichiro Maeshima, Hiroya Koshiba, Wataru Sakamoto

**Affiliations:** 1 Department of Health and Sport Management, Osaka University of Health and Sport Sciences, 1-1 Asashirodai, Kumatori-cho, Sennan-gun, Osaka, 590-0496 Japan; 2 Third Department of Internal Medicine, Wakayama Medical University, Wakayama, Japan; 3 Department of Rehabilitation Medicine, International Medical Center, Saitama Medical University, Saitama, 350-1298 Japan; 4 Serotec Laboratory, Hokkaido, 069-0822 Japan

**Keywords:** Autoimmune diseases, Salivary gland, Hyposalivation, Screening technique

## Abstract

We have investigated the prevalence of dry mouth among patients with autoimmune diseases other than Sjögren’s syndrome. One hundred and forty-four patients, excluding patients with primary Sjögren’s syndrome, were enrolled in this study. The volume of saliva secreted was measured with the screening technique for estimation of salivary flow, which uses a filter paper for diagnosing dry mouth. Disturbed salivary secretion was observed in 84 (58.3 %) of the 144 patients. In the case of patients free of Sjögren’s syndrome, the prevalence of disturbed salivary secretion differed significantly among the disease groups (*P* < 0.05), with the prevalence being over 50 % in all disease groups other than the rheumatoid arthritis group and the highest in the systemic sclerosis group. There was significant positive correlation between the number of colored spots and oral visual analog scale score (*r* = 0.45, *P* < 0.0001). Autoimmune diseases can be accompanied by salivary gland dysfunction, regardless of the presence/absence of complication by Sjögren’s syndrome. In the present study, the screening technique for estimation of salivary flow, which uses a filter paper for diagnosing dry mouth, was shown to be a useful means of detecting salivary gland dysfunction.

## Introduction

Dry mouth (xerostomia) is a condition caused by reduced salivary secretion (<100 μl/min) [1]. It can be caused by various factors such as mental/physical stress, sickness, and medication. It is characterized by inflammation of the tongue and oral mucosa, leading to anorexia/reduced appetite (due to pain), foul breath, stomatitis, and dental caries which reduce the quality of life and elevate susceptibility to infection. For this reason, early detection/diagnosis of dry mouth is essential for maintaining and promoting systemic and oral health. A representative autoimmune disease likely to be complicated by dry mouth is Sjögren’s syndrome (SjS). The prevalence of dry mouth in association with other autoimmune diseases is unknown. The diagnosis of dry mouth is based on a minor salivary gland biopsy of the lips, gum test, Saxon test, sialography, and scintigraphy. These tests, however, require complex manipulation and take long time for diagnosis. In addition, they are invasive. For these reasons, these tests are not often used for screening of dry mouth. Kanehira et al. [2] reported the screening technique for estimation of salivary flow (STESF), which uses a filter paper for diagnosing dry mouth. This technique enables simple, rapid, and noninvasive diagnosis of dry mouth by chairside assessments. The present study was undertaken to investigate the prevalence of dry mouth among patients with autoimmune diseases other than SjS. Thus, we compared the amount of salivary secretion in patients with various autoimmune diseases, as measured with the filter paper for the diagnosis of dry mouth, with that in patients with SjS.

## Subjects and methods

Of the 161 patients with collagen disease who visited the Outpatient Collagen Disease Clinic of Wakayama Medical University Hospital between July 2000 and February 2011, 144 patients, excluding patients with primary Sjögren’s syndrome, were enrolled in the study. The diagnosis was rheumatoid arthritis (RA) in 56 patients, systemic lupus erythematosus (SLE) in 46 patients, polymyositis/dermatomyositis (PM/DM) in 8 patients, systemic scleroderma (SSc) in 26 patients and mixed connective tissue disease (MCTD) in 8 patients. Complication by secondary Sjögren’s syndrome (SjS) was observed in 46 patients (14 patients with RA, 25 with SLE, 1 with PM/DM, 2 with SSc, and 4 with MCTD). In these subjects, the volume of saliva secreted was measured with STESF. In this test, the color developer solution was dropped onto the filter paper inserted for 2 min into the sublingual area. The number of brown to blue spots was counted. An increase in the number of colored spots indicates less saliva secretion. If the volume of saliva secretion at rest is 200 μl/min or lower, the diagnosis is salivary gland dysfunction [3], and if the volume is 100 μl/min or lower, the diagnosis is dry mouth [1]. In these patients, the number of colored spots will be 3 and 4, respectively [2]. Hence, patients with 3 or more colored spots were diagnosed with disturbed salivary secretion in this study. Furthermore, each patient rated the subjective sensation of oral dryness on a visual analog scale (oral VAS). The relationship between VAS score and the number of colored spots was analyzed. Each subject was informed about the purpose and methods of the study, and written consent to participate in the study was obtained in advance. Each subject was instructed to practice brushing 2 h before the test, and subsequent food intake was not allowed. Water intake was prohibited during the 1-h period before the test.

### Statistical analysis

Chi-square test was used for comparing the presence/absence of disturbed salivary secretion among the different disease groups. Spearman’s rank correlation coefficient was used for analyzing the association between the number of colored spots and oral VAS score. A* p* value less than 0.05 was considered statistically significant.

## Results

**Fig. 1:**
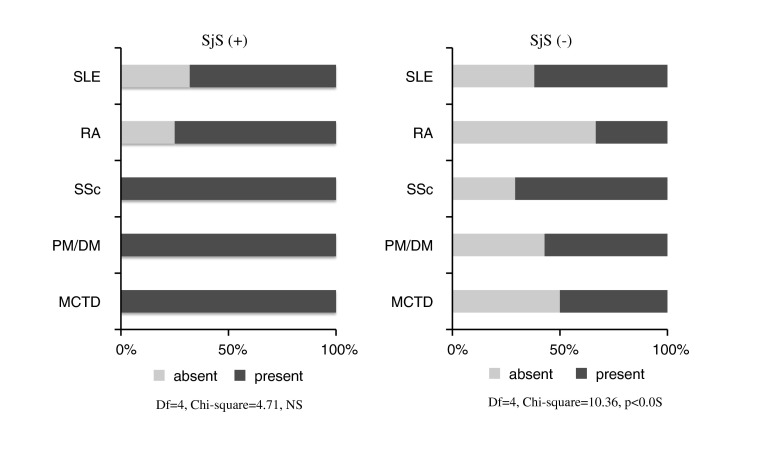
In the case of patients with SjS, the prevalence of disturbed salivary secretion did not differ depending on the underlying disease, and its prevalence was high in all disease groups. In the case of patients free of SjS, the prevalence of disturbed salivary secretion differed significantly among the disease groups (*P* < 0.05), with the prevalence being over 50 % in all disease groups other than the RA group and the highest in the SSc group

Disturbed salivary secretion was observed in 84 (58.3 %) of the 144 patients. Each disease group was further subdivided into the SjS-complicated group and the SjS-free group. The presence/absence of disturbed salivary secretion was compared between these 2 subgroups. In the case of patients with SjS, the prevalence of disturbed salivary secretion did not differ depending on the underlying disease, and its prevalence was high in all disease groups. In the case of patients free of SjS, the prevalence of disturbed salivary secretion differed significantly among the disease groups (*P* < 0.05), with the prevalence being over 50 % in all disease groups other than the RA group and the highest in the SSc group (Fig. 1).

**Fig. 2:**
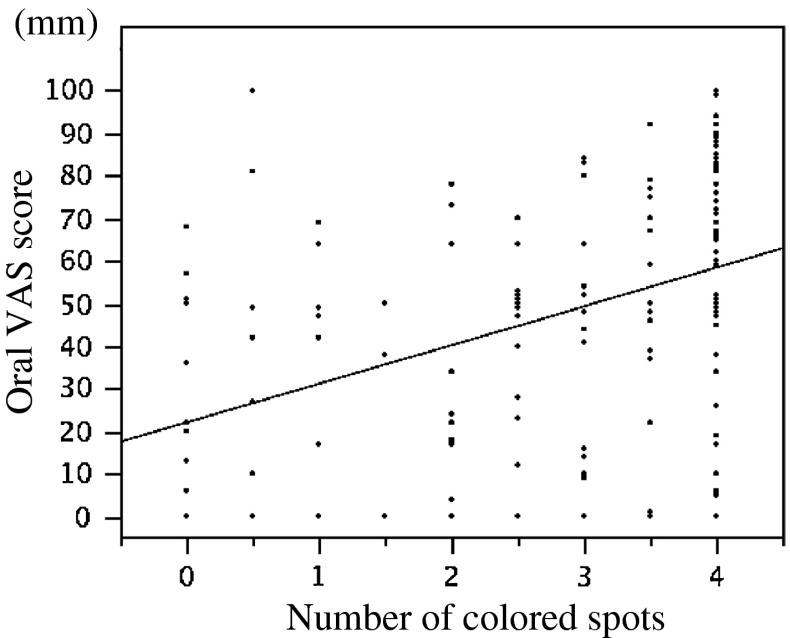
There was significant positive correlation between the number of colored spots and oral VAS score (*r* = 0.45, *P* < 0.0001), indicating that the volume of saliva secreted decreased with increase in the subjective sensation of oral dryness

There was significant positive correlation between the number of colored spots and oral VAS score (*r* = 0.45, *P* < 0.0001), indicating that the volume of saliva secreted decreased with increase in the subjective sensation of oral dryness (Fig. 2).

## Discussion

It has been pointed out that the hygiene status within the oral cavity is associated with various diseases, including cardiovascular disease and airway infection [4]. Recently, reports on association of periodontal disease with the onset, progression, and severity of autoimmune disease have been published sporadically [4–9]. Cardiovascular disease and airway infection are some of the main causes of death in patients with autoimmune disease [9–12]. The hygiene status of the oral cavity may have a large influence on the onset of autoimmune disease and the causes of death among patients with autoimmune disease. Bearing these previous reports in mind, we measured the volume of saliva secreted with STESF to assess the hygiene status of the oral cavity of individual patients in the present study.

In patients with SjS, mononuclear cells invaded the salivary gland and the subsequent inflammation destroyed the salivary gland, reducing the volume of saliva secreted [13]. In the present study, however, disturbed salivary secretion was noted frequently even in SjS-free patients. Considering the previous report that SSc histologically involved fibrous changes of the salivary gland [14, 15], it appears likely that the mechanism for decrease in salivary secretion observed in patients with SSc differs from that in patients with SjS. Patients with MCTD presented with SSc-like clinical symptoms, suggesting the presence of histological changes in the salivary gland akin to SSc. However, the exact mechanism for development of disturbed salivary secretion in patients with other autoimmune diseases remains unknown, and studies based on salivary gland biopsy and radiological techniques are needed to resolve this question.

Advancement of SSc increases the severity of trismus (due to facial skin sclerosis) and flexion contracture of fingers, making brushing and dental manipulation difficult. Rheumatologists dealing with these cases are required to take into account the possibility of disturbed salivary secretion, regardless of the presence or absence of SjS, and to begin oral care at early stages in cooperation with nurses, dentists, oral surgeons, and others.

In addition, the present study revealed close correlation between the number of colored spots and oral VAS score. There is no previous report dealing with the relationship between the volume of saliva secreted (measured with STESF) and the subjective sensation of oral dryness. The results from the present study indicate that STESF is a useful screening test for objective evaluation of oral dryness.

## Conclusion

Autoimmune diseases can be accompanied by salivary gland dysfunction, regardless of the presence/absence of complication by SjS. In the present study, STESF was shown to be a useful means of detecting salivary gland dysfunction.
